# Single-center clinical laboratory evaluation of VITEK REVEAL for rapid phenotypic antimicrobial susceptibility testing of Gram-negative organisms directly from positive blood cultures

**DOI:** 10.3389/fmicb.2026.1890096

**Published:** 2026-08-05

**Authors:** Yuan Chao Xue, Natalie Williams-Bouyer, Ping Ren

**Affiliations:** Department of Pathology, University of Texas Medical Branch at Galveston, Galveston, TX, United States

**Keywords:** clinical comparative study, Gram-negative bloodstream infections, rapid phenotyping antimicrobial susceptibility testing, rapid susceptibility test, VITEK REVEAL system

## Abstract

**Objectives:**

Rapid phenotypic antimicrobial susceptibility testing (AST) has the potential to support earlier optimization of antimicrobial therapy for patients with bloodstream infections. We evaluated the real-world clinical laboratory performance of the bioMérieux VITEK REVEAL® rapid phenotypic AST system using positive blood cultures containing Gram-negative (GN) organisms and compared its results with those obtained using the routine standard-of-care (SOC) MicroScan WalkAway Plus system.

**Methods:**

A total of 200 positive blood culture bottles yielding GN organisms were evaluated. VITEK REVEAL® performance was assessed using categorical agreement (CA), essential agreement (EA), and error rates, including very major errors (VMEs), major errors (MEs), and minor errors (mEs), with the MicroScan serving as the comparator method. The Etest was performed as a supplemental exploratory method to evaluate selected VME and ME discrepancies.

**Results:**

Within the local resistance epidemiology represented in this study, VITEK REVEAL showed ≥90% EA across evaluated antimicrobial–organism combinations and ≥90% CA for all combinations except amoxicillin/clavulanate (AMC) and ampicillin/sulbactam (SAM). Across the 2,536 antimicrobial–organism combinations evaluated, 14 VMEs (0.6%), 16 MEs (0.6%), and 82 mEs (3.3%) were observed. Several elevated error rates were associated with limited numbers of resistant isolates and breakpoint-adjacent minimum inhibitory concentration (MIC) distributions. Supplemental Etest analysis reduced the number of selected discrepancies, although reference broth microdilution was not performed.

**Conclusion:**

This single-center study was designed as a real-world comparative clinical laboratory evaluation and is limited by the use of an automated comparator method rather than reference broth microdilution, the local resistance epidemiology, and limited isolate numbers for certain antimicrobial–organism combinations. By providing phenotypic AST results directly from positive blood cultures in less than 8 h, VITEK REVEAL may support earlier therapeutic decision-making and antimicrobial stewardship efforts. Additional multicenter and clinical outcome studies are needed to better determine its clinical impact.

## Introduction

Bloodstream infections (BSIs) and their associated sepsis are life-threatening conditions and major causes of morbidity and mortality worldwide (Singer et al., 2016). Sepsis is associated with increased mortality, prolonged hospitalization, and increased intensive care unit (ICU) utilization (Paoli et al., 2018). Early initiation of appropriate antimicrobial therapy is critical for improving patient outcomes. However, the increasing prevalence of multidrug-resistant (MDR) bacterial pathogens has substantially limited available treatment options and remains a major challenge in infectious disease management (van Duin and Paterson, 2016; Ohnuma et al., 2023; Webb et al., 2019; Tan et al., 2022).

Rapid and reliable phenotypic antimicrobial susceptibility testing (AST) is therefore essential to support timely targeted therapy (van Belkum et al., 2020; Maurer et al., 2017; Doern, 2018; Galar et al., 2012). Conventional AST workflows typically require bacterial isolation from positive blood culture bottles prior to susceptibility testing, resulting in delays of approximately 16 h or more before the final AST results become available. During this interval, patients with suspected BSIs, sepsis, or septic shock are usually treated with empiric broad-spectrum antimicrobial therapy. Although often necessary, prolonged empiric therapy may increase adverse drug effects, promote the selection of resistant organisms, and contribute to suboptimal antimicrobial stewardship practices (Salam et al., 2023; Gajic et al., 2022; Jacobs et al., 2021; Llor and Bjerrum, 2014; Muteeb et al., 2023). Consequently, there is substantial clinical interest in rapid phenotypic AST methods capable of providing earlier susceptibility results directly from positive blood cultures.

The bioMérieux VITEK REVEAL® phenotyping AST system (VITEK REVEAL®), formerly the REVEAL® Rapid AST System (Specific Diagnostics, USA), is designed to generate phenotypic AST results directly from positive blood cultures in less than 8 h. The system received U.S. Food and Drug Administration (FDA) clearance in 2024 for Gram-negative (GN) bacteria recovered from positive blood cultures (bioMérieux, 2024). VITEK REVEAL® uses chemical sensors to detect volatile metabolic compounds produced during bacterial growth in the presence of antimicrobial agents at various concentrations, enabling rapid determination of susceptibility profiles (Flentie et al., 2019). By reducing AST turnaround time (TAT), this technology may support the earlier optimization of antimicrobial therapy. However, independent real-world clinical laboratory evaluations following FDA clearance remain limited.

In this study, we conducted a single-center clinical laboratory evaluation of the VITEK REVEAL® system using positive blood cultures yielding GN organisms and compared the results with those generated by the routine standard-of-care MicroScan WalkAway Plus microbiology system (MicroScan). This study aimed to assess the comparative performance and operational feasibility of implementing the VITEK REVEAL® system in a real-world clinical laboratory workflow.

## Methods

### Study design and isolates

**Figure 1:**
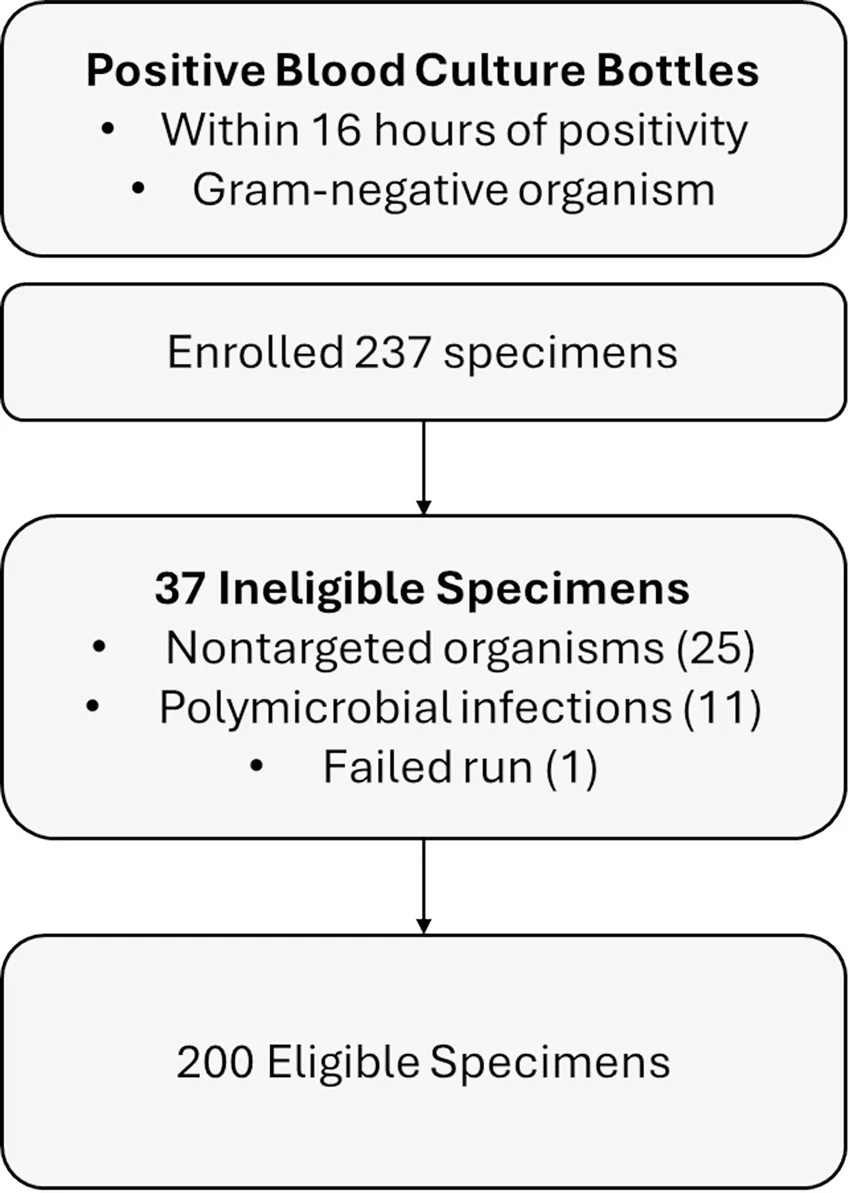
Specimen enrollment workflow.

A total of 237 consecutive positive blood culture bottles yielding GN organisms were collected from the Clinical Microbiology Laboratory at our institution between November 2024 and October 2025 (Figure 1). Testing was performed in parallel with the routine clinical microbiology workflow. Isolates were recovered from positive blood culture bottles within 16 h of positivity, in accordance with the manufacturer’s recommendations. Organism identification and antimicrobial susceptibility testing were subsequently performed using the conventional culture-based workflow—the MicroScan WalkAway Plus AST system—described in the following section.

### MicroScan WalkAway plus AST system

**Figure 2:**
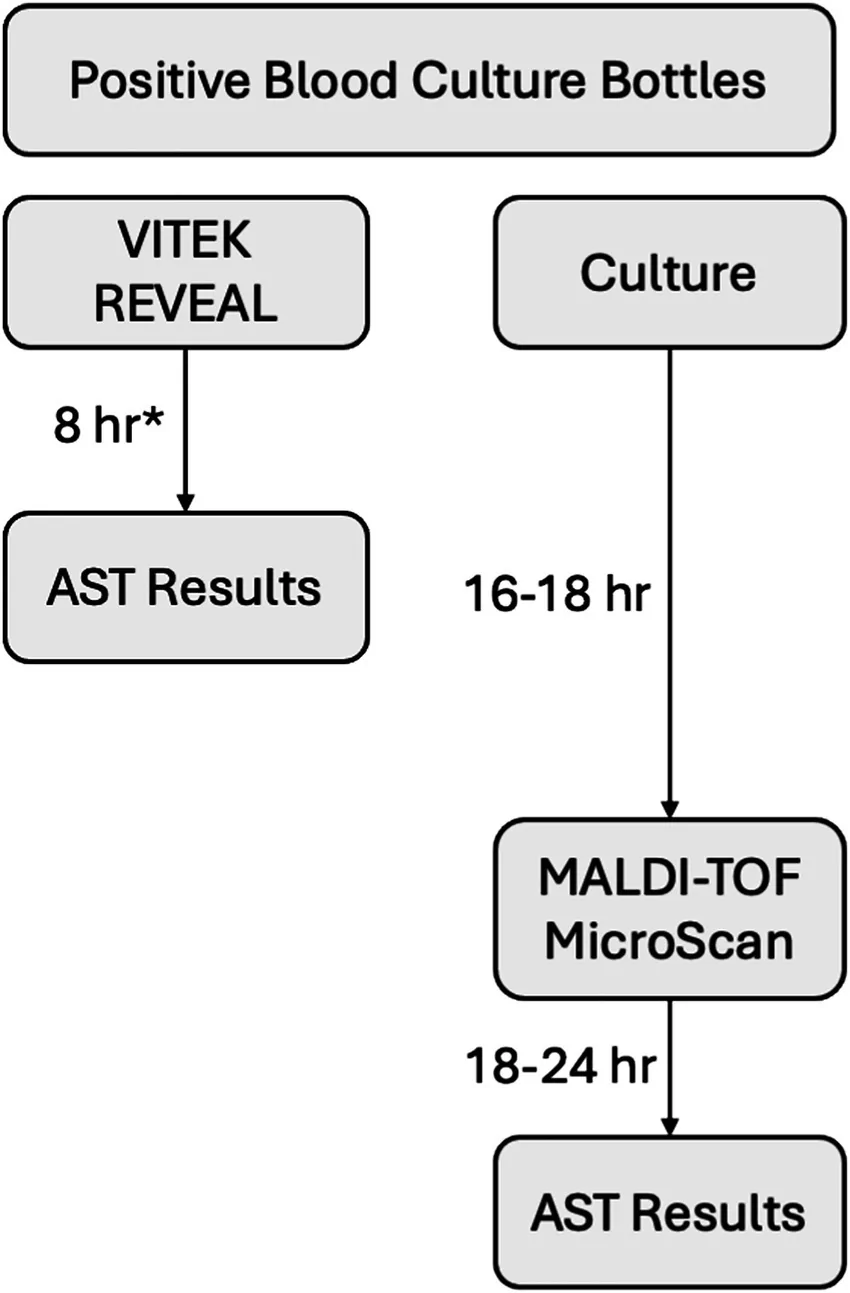
MicroScan and VITEK REVEAL® workflow.\r*If organism identification is available within 8 h.

After 16–18 h of incubation on 5% sheep blood agar (SBA) (Remel, Lenexa, KS, USA), isolates were identified using matrix-assisted laser desorption/ionization time-of-flight mass spectrometry (MALDI-TOF MS) (Bruker MALDI Biotyper CA, version 3.2, with the MALDI Biotyper CA library) (Figure 2). AST was performed using the MicroScan NM56 Panels (Beckman Coulter, Brea, CA, USA), which served as the routine standard-of-care (SOC) comparator system (Figure 2).

### VITEK REVEAL® AST system

Positive blood culture bottles demonstrating GN organisms by Gram stain were processed as soon as possible following positivity detection or after the Verigene Gram-Negative Blood Culture Test (Diasorin, Austin, TX, USA) results became available, as long as processing occurred within 16 h of positivity in accordance with the manufacturer’s instructions (Figure 2). Organism identification was confirmed using the MALDI-TOF MS results. Briefly, 2–3 mL of positive blood culture broth was transferred to a sterile tube, and a 20-μL aliquot was diluted in 10 mL of Pluronic inoculum water (BD, Franklin Lakes, NJ, USA). The diluted suspension was inoculated into a BC GN02-AST FDA-cleared panel (bioMérieux, Durham, NC, USA) using a Renok tray and a Renok rehydrator (BD, Franklin Lakes, NJ, USA). A VITEK REVEAL® sensor panel (bioMérieux, Durham, NC, USA) was sealed onto each AST panel using the VITEK REVEAL® plate sealer (bioMérieux, Durham, NC, USA).

For purity control, an aliquot of the inoculum mixture from the Renok tray was subcultured onto 5% SBA and incubated for 18–24 h at 35 °C in ambient air. Culture purity was assessed by visual inspection for consistent colony morphology. Bacterial identifications were entered into the VITEK REVEAL® software. MICs were then generated and interpreted according to the 2025 Clinical and Laboratory Standards Institute (CLSI) breakpoints. Manufacturer-recommended quality control (QC) testing was performed using *Escherichia coli* ATCC 25922 to verify assay reproducibility and system performance with each new reagent lot, following instrument or software modifications, or when internal QC results failed (bioMérieux, Durham, NC, USA).

The BC GN02 AST FDA-cleared panel (bioMérieux, Durham, NC, USA) includes 16 antimicrobial agents: amoxicillin/clavulanate (AMC), ampicillin/sulbactam (SAM), piperacillin/tazobactam (TZP), ceftazidime (CAZ), ceftriaxone (CRO), cefepime (FEP), ceftolozane/tazobactam (CFT), ceftazidime/avibactam (CZA), ertapenem (ETP), meropenem (MEM), imipenem (IMP), meropenem/vaborbactam (MEV), ciprofloxacin (CIP), levofloxacin (LVX), tobramycin (TOB), and trimethoprim/sulfamethoxazole (SXT). Only FDA-cleared and reportable antimicrobial–organism combinations were included in the analysis. Target organisms for the panel include the *Acinetobacter baumannii* complex, *Citrobacter freundii* complex, *Citrobacter koseri*, *Escherichia coli*, *Enterobacter cloacae* complex, *Klebsiella oxytoca*, *Klebsiella pneumoniae*, *Klebsiella aerogenes*, *Pseudomonas aeruginosa*, *Proteus mirabilis*, *Proteus vulgaris*, and *Serratia marcescens*.

### Exploratory Etest analysis

The Etest (bioMérieux, Durham, NC, USA) was performed as a supplementary exploratory analysis for selected isolates demonstrating VME or ME discrepancies between the two testing systems. Frozen isolates were subcultured twice onto 5% SBA and incubated for 18–24 h at 35 °C in ambient air. A 0.5 McFarland suspension was prepared in saline and inoculated onto Mueller–Hinton agar (Remel, Lenexa, KS, USA). Etest strips were applied, and plates were incubated for 18–24 h at 35 °C in ambient air prior to MIC interpretation according to the manufacturer’s instructions.

### Data analysis

Since reference broth microdilution (BMD) was not performed, analyses were intended to assess comparative agreement between VITEK REVEAL® and the routine SOC AST workflow rather than to establish definitive analytical performance characteristics.

MIC values and categorical interpretations generated by the VITEK REVEAL system were compared with those obtained using the SOC MicroScan system. CA and EA were calculated for each available antimicrobial–organism combination (Supplementary Table S1) (bioMérieux, 2026). Both systems used the 2025 CLSI M100/FDA breakpoints that were in effect during the study period. CA was defined as agreement in categorical interpretation between VITEK REVEAL and the SOC MicroScan system following application of CLSI/FDA breakpoints. EA was defined as an MIC agreement within ±1 doubling dilution between the two testing systems.

Since both systems reported MICs using predefined dilution ranges, off-scale MIC handling criteria adapted from the manufacturer’s FDA submission documentation were applied for comparative EA analysis:

1. MIC values reported as ≤ X or ≥ X were assigned a value of X.
2. MIC values reported as > X were assigned a value of one doubling dilution above X.
3. When both systems reported MICs at their lowest or highest measurable value, the results were considered concordant for comparative EA analysis.
4. When one system reported ≤ X and the other reported a value below X, the results were considered concordant.

VMEs, MEs, and mEs were defined according to the CLSI criteria (CLSI, 2023). A VME was defined as an isolate categorized as resistant by the comparator system but susceptible by VITEK REVEAL®. An ME was defined as an isolate categorized as susceptible by the comparator system but resistant by VITEK REVEAL®. An mE was defined as a discrepancy involving an intermediate (I) or susceptible dose-dependent (SDD) category.

Error rates were calculated as follows:

- VME rate (%) = (number of VMEs/number of resistant isolates by the comparator system) × 100.
- ME rate (%) = (number of MEs/number of susceptible isolates by the comparator system) × 100.
- mE rate (%) = (number of mEs/total number of isolates tested) × 100.

Following supplementary Etest analysis, categorical interpretations for selected VME and ME discrepancies were compared descriptively. For isolates with VMEs, an Etest categorical interpretation of susceptible was considered concordant with the VITEK REVEAL categorical interpretation, whereas a resistant Etest interpretation was considered concordant with the MicroScan categorical interpretation. For isolates with MEs, an Etest categorical interpretation of resistant was considered concordant with the VITEK REVEAL categorical interpretation, whereas a susceptible Etest interpretation was considered concordant with the MicroScan categorical interpretation. Isolates yielding an intermediate/susceptible dose-dependent isolates (I/SDD) Etest interpretation were excluded from supplementary discrepancy comparisons because these results did not preferentially align with either system. Etest results were used for supplementary descriptive comparison only and not as a reference adjudication method.

## Results

Of the 237 specimens included in the study, 37 were excluded from the final analysis (Figure 1). Specifically, 25 specimens produced organisms that did not meet the FDA-cleared panel criteria, 11 were polymicrobial blood cultures, and 1 had an instrument failure. Among the 200 GN clinical isolates included in the analysis, the most frequently identified organisms were *Escherichia coli* (94 isolates, 47.0%), *Klebsiella pneumoniae* (37 isolates, 18.5%), and *Proteus mirabilis* (13 isolates, 6.5%) (Table 1).

**Table 1:** List of clinical Gram-negative organisms from positive blood culture bottles.

Organisms	Number isolates	Percentage
*Escherichia coli*	94	47.0%
*Klebsiella pneumoniae*	37	18.5%
*Proteus mirabilis*	13	6.5%
*Serratia marcescens*	10	5.0%
*Enterobacter cloacae* complex	10	5.0%
*Klebsiella oxytoca*	3	1.5%
*Klebsiella aerogenes*	2	1.0%
*Citrobacter freundii* complex	2	1.0%
*Proteus vulgaris*	1	0.5%
*Pseudomonas aeruginosa*	24	12.0%
*Acinetobacter baumannii*	4	2.0%
Total	200	

Organism identification was confirmed using the Verigene Gram-Negative Blood Culture Test, which required 2–3 h. The mean turnaround time (TAT) for VITEK REVEAL AST was 7 h and 24 min ± 44 min. In comparison, the mean TAT for the MicroScan system was 25 h 14 min ± 6 h and 11 min, in addition to approximately 16–18 h required for standard subculture recovery from positive blood bottles prior to AST setup (Figure 2).

**Figure 3:**
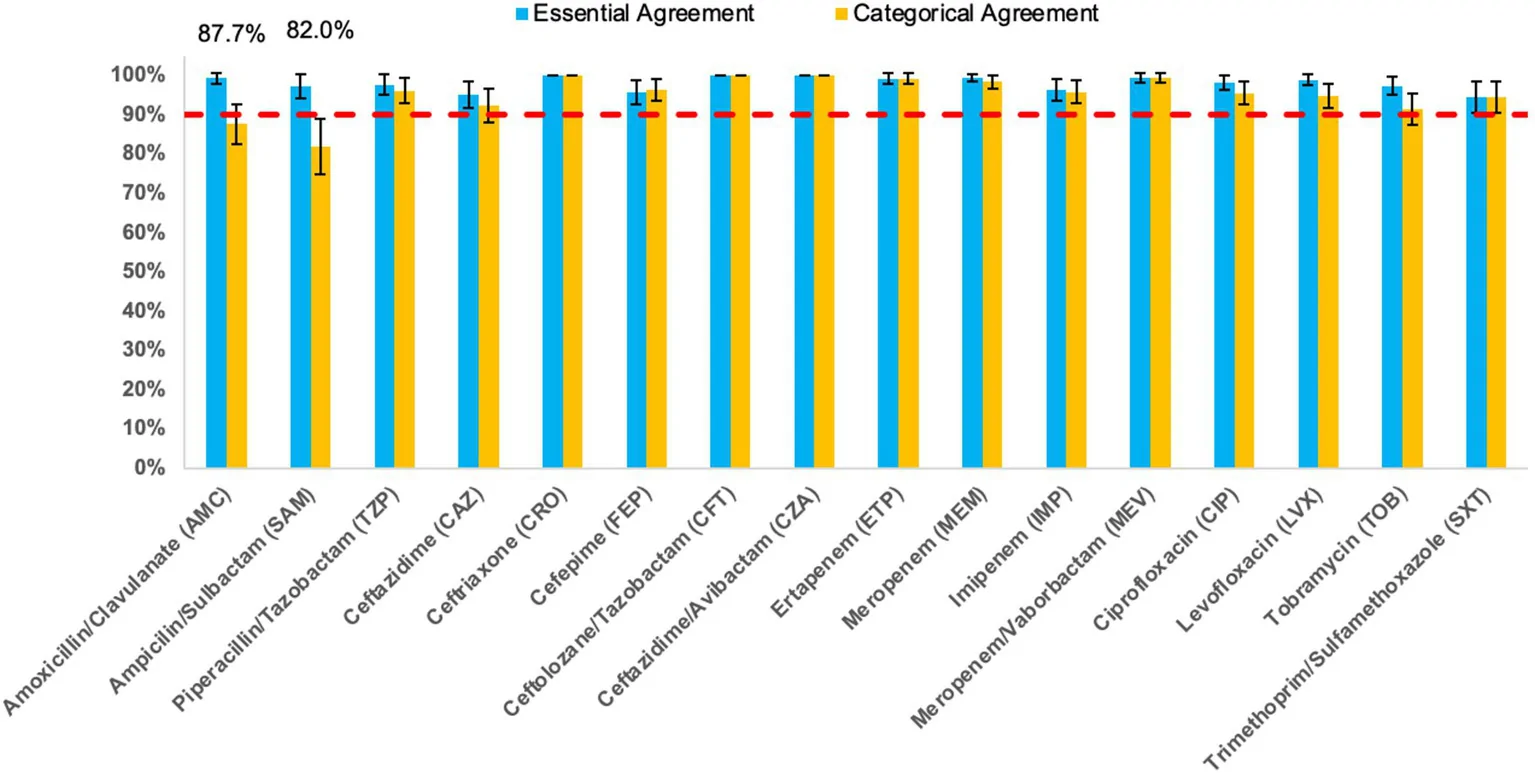
Essential and categorical agreement between VITEK REVEAL® and MicroScan WalkAway System AST results on Gram-negative organisms from positive blood cultures. All values are shown in percentages (%). The red dashed line illustrates CLSI’s 90% CA and EA cutoff. The black line illustrates the corresponding 95% confidence intervals.

Across all evaluated antimicrobial–organism combinations, overall EA was 98.1% and CA was 95.5%. CA for individual antimicrobial agents was ≥ 90% for all agents except amoxicillin/clavulanate (87.7%, 135/154) and ampicillin/sulbactam (82.0%, 91/111) (Figure 3).

Among the 2,536 antimicrobial–organism combinations evaluated, 114 categorical discrepancies (4.5%) were identified, including 14 VMEs (0.55%), 16 MEs (0.63%), and 82 mEs (3.3%) (Table 2). Antimicrobial–organism combinations with VME and ME rates >3% or mE rates >10%, exceeding CLSI/FDA performance thresholds, are highlighted in bold in Table 2. Tobramycin accounted for the highest number of VMEs (4 of 14 total VMEs) among Enterobacterales. Trimethoprim/sulfamethoxazole accounted for the highest number of MEs (4 of 16 total MEs), whereas amoxicillin/clavulanate and ampicillin/sulbactam accounted for the highest numbers of mEs (18 of 82 and 20 of 82, respectively).

**Table 2:** List of categorical discrepancies.

Antimicrobial agents^4^	Organisms^4^	Number of isolates based on MicroScan MIC^1^				VME^1^		ME^1^		mE^1^	
		S^1^	I/SDD^1^	R^1^	T^1^	#^2^	%^2^	#	%	#	%
Amoxicillin/clavulanate (AMC)	Enterobacterales (EC, KO, KP, PM)^3^	120	21	13	154	0	0.0%	1	0.8%	18	**11.7%**
Ampicillin/sulbactam (SAM)	Enterobacterales (EC, KO, PM)	58	29	24	111	0	0.0%	0	0.0%	20	**18.0%**
Piperacillin/tazobactam (TZP)	Enterobacterales (EC, KP, PV)	126	1	4	131	2	**50.0%**	1	0.8%	2	1.5%
Ceftazidime (CAZ)	Enterobacterales (ECC, EC, KA, KO, KP)	109	1	32	142	2	**6.3%**	1	0.9%	6	4.2%
	*Acinetobacter baumannii*	2	1	1	4	0	0.0%	0	0.0%	0	0.0%
Ceftriaxone (CRO)	Enterobacterales (ECC, EC, KA, KO, KP, PM)	118	0	37	155	0	0.0%	0	0.0%	0	0.0%
Cefepime (FEP)	Enterobacterales (ECC, EC, KA, KO, KP)	109	1	35	145	1	2.9%	0	0.0%	5	3.4%
	*Pseudomonas aeruginosa*	20	0	3	23	0	0.0%	0	0.0%	0	0.0%
Ceftolozane/tazobactam (CFT)	Enterobacterales (ECC, EC, KO, PM)	113	0	0	113	NE	NE^2^	0	0.0%	0	0.0%
	*Pseudomonas aeruginosa*	23	0	0	23	NE	NE	0	0.0%	0	0.0%
Ceftazidime/avibactam (CZA)	Enterobacterales (ECC, EC, KA, KP, PM, CFC)	150	0	0	150	NE	NE	0	0.0%	0	0.0%
	*Pseudomonas aeruginosa*	23	0	0	23	NE	NE	0	0.0%	0	0.0%
Ertapenem (ETP)	Enterobacterales (EC, KP, PM, PV, CFC)	145	1	0	146	NE	NE	1	0.7%	0	0.0%
Meropenem (MEM)	Enterobacterales (ECC, EC, KP, PM, PV, SM)	165	0	0	165	NE	NE	1	0.6%	1	0.6%
	*Pseudomonas aeruginosa*	19	1	3	23	0	0.0%	0	0.0%	1	4.3%
	*Acinetobacter baumannii*	2	0	2	4	0	0.0%	0	0.0%	0	0.0%
Imipenem (IMP)	Enterobacterales (ECC, EC, KO, KP, SM)	142	2	0	144	NE	NE	2	1.4%	3	2.1%
	*Pseudomonas aeruginosa*	18	1	4	23	0	0.0%	0	0.0%	2	8.7%
	*Acinetobacter baumannii*	2	0	2	4	0	0.0%	0	0.0%	0	0.0%
Meropenem/vaborbactam (MEV)	Enterobacterales (ECC, EC, KA, KO, KP, PM)	160	0	0	160	NE	NE	1	0.6%	0	0.0%
Ciprofloxacin (CIP)	Enterobacterales (ECC, EC, KA, KO, KP, PV, SM, CFC)	112	3	43	158	1	2.3%	3	2.7%	3	1.9%
	*Pseudomonas aeruginosa*	19	0	4	23	0	0.0%	0	0.0%	1	4.3%
Levofloxacin (LVX)	Enterobacterales (ECC, EC, KA, KO, KP, PM, PV, SM, CFC)	129	4	39	172	1	2.6%	1	0.8%	6	3.5%
	*Pseudomonas aeruginosa*	17	2	4	23	0	0.0%	0	0.0%	2	8.7%
Tobramycin (TOB)	Enterobacterales (ECC, EC, KA, KO, KP, PM, SM, CFC)	139	8	24	171	4	**16.7%**	0	0.0%	12	7.0%
	*Pseudomonas aeruginosa*	17	0	0	17	NE	NE	0	0.0%	0	0.0%
Trimethoprim/sulfamethoxazole (SXT)	Enterobacterales (EC, KA, KP)	82	0	47	129	3	**6.4%**	4	**4.9%**	0	0.0%
Total		2,139	76	321	2,536	14		16		82	

1 VME, very major error (# of VMEs/# of R isolates based on MicroScan); ME, major error (# of MEs/# of S isolates based on MicroScan); mE, minor error (# of mEs/# of total isolates based on MicroScan); S, susceptible isolates; I/SDD, intermediate/susceptible dose-dependent isolates; R, resistant isolates; T, total isolates; MIC, minimal inhibitory concentration.

2 #, the number of isolates; %, error rate percentages for VME, ME, and mE of each antimicrobial–organism combination as described in the Materials and methods section; NE, not evaluable where there is 0 isolate in the appropriate denominator.

3 The Enterobacterales group includes species: CFC, *Citrobacter freundii* complex; ECC, *Enterobacter cloacae* complex; EC, *Escherichia coli*; KA*, Klebsiella aerogenes*; KO, *Klebsiella oxytoca*; KP, *Klebsiella pneumoniae*; PM, *Proteus mirabilis*; PV, *Proteus vulgaris*; SM, *Serratia marcescens*.

4 Not all species are evaluated using all available antimicrobial agents. See Supplementary Table S1 for the evaluated antimicrobial–organism combination.

Antimicrobial–organism combinations with VME and ME rates >3% or mE rates >10%, exceeding CLSI/FDA performance thresholds, are highlighted in bold.

Supplementary Etest analysis was performed for selected VME and ME discrepancies involving corresponding antimicrobial–organism combinations (Tables 3, 4). Among isolates with VMEs, Etest categorical interpretations were concordant with VITEK REVEAL® in 5 of 14 cases, concordant with MicroScan in 6 of 14 cases, and discordant with both systems in 3 of 14 cases. Among isolates with MEs, Etest categorical interpretations were concordant with VITEK REVEAL in 1 of 16 cases, concordant with MicroScan in 13 of 16 cases, and discordant with both systems in 2 of 16 cases.

**Table 3:** Etest results for enterobacterales antimicrobial–organism combinations with very major and major errors.

Antibiotics	Organisms	VME^1^ Etest result				ME^1^ Etest result			
		Isolate #^1^	S^1^	I/SDD^1^	R^1^	Isolate #^1^	S^1^	I/SDD^1^	R^1^
Amoxicillin/clavulanate (AMC)	*Escherichia coli*	0				1		1	
Piperacillin/tazobactam (TZP)	*Escherichia coli, Klebsiella pneumoniae*	2		2		1		1	
Ceftazidime (CAZ)	*Escherichia coli, Klebsiella aerogenes*	2	1	1		1	1		
Cefepime (FEP)	*Escherichia coli*	1	1			0			
Ertapenem (ETP)	*Escherichia coli*	0				1	1		
Meropenem (MEM)	*Escherichia coli*	0				1	1		
Imipenem (IMP)	*Escherichia coli, Klebsiella pneumoniae*	0				2	2		
Meropenem/vaborbactam (MEV)	*Escherichia coli*	0				1	1		
Ciprofloxacin (CIP)	*Escherichia coli, Serratia marcescens, Klebsiella pneumoniae*	1			1	3	2		1
Levofloxacin (LVX)	*Escherichia coli, Serratia marcescens*	1			1	1	1		
Tobramycin (TOB)	*Escherichia coli, Klebsiella pneumoniae*	4	3		1	0			
Trimethoprim/sulfamethoxazole (SXT)	*Escherichia coli, Klebsiella pneumoniae*	3			3	4	4		
Total		14	5	3	6	16	13	2	1

1 VME, very major error; ME, major error; S, susceptible isolates; I/SDD, intermediate/susceptible dose-dependent isolates; R, resistant isolates; #, the number of isolates tested.

2Green columns indicate Etest results favoring VITEK REVEAL®. Red columns indicate results favoring MicroScan.

**Table 4:** Comparison of very major and major error rates by antimicrobial–organism combination before and after Etest evaluation.

			Isolate # (MicroScan)^2^				VME^1^		ME^1^		mE^1^	
Abbreviation	Organisms	Etest	S^1^	I/SDD^1^	R^1^	T^1^	#	%^1^	#	%	#	%
Amoxicillin/Clavulanate (AMC)	Enterobacterales (EC, KO, KP, PM)^3^	Before	120	21	13	154	0	0.0%	1	0.8%	18	11.8%
		After	119	21	13	153	0	0.0%	0	0.0%	18	11.8%
Piperacillin/Tazobactam (TZP)	Enterobacterales (EC, KP, PV)	Before	126	1	4	131	2	50.0%	1	0.8%	2	1.5%
		After	125	1	2	128	0	0.0%	0	0.0%	2	1.5%
Ceftazidime (CAZ)	Enterobacterales (ECC, EC, KA, KO, KP)	Before	109	1	32	142	2	6.3%	1	0.9%	6	4.2%
		After	109	1	31	141	0	0.0%	1	0.9%	6	4.2%
Cefepime (FEP)	Enterobacterales (ECC, EC, KA, KO, KP)	Before	109	1	35	145	1	2.9%	0	0.0%	5	3.4%
		After	109	1	35	145	0	0.0%	0	0.0%	5	3.4%
Ciprofloxacin (CIP)	Enterobacterales (ECC, EC, KA, KO, KP, PV, SM, CFC)	Before	112	3	43	158	1	2.3%	3	2.7%	3	1.9%
		After	112	3	43	158	1	2.3%	2	1.8%	3	1.9%
Tobramycin (TOB)	Enterobacterales (ECC, EC, KA, KO, KP, PM, SM, CFC)	Before	139	8	24	171	4	16.7%	0	0.0%	12	7.0%
		After	139	8	24	171	1	4.2%	0	0.0%	12	7.0%

1 VME, very major error (# of VMEs/# of R isolates based on MicroScan); ME, major error (# of MEs/# of S isolates based on MicroScan); mE, minor error (# of mEs/# of total isolates based on MicroScan); S, susceptible isolates; I/SDD, intermediate/susceptible dose-dependent isolates; R, resistant isolates; T, total isolates.

2 #, the number of isolates; %, error rate percentages for VME, ME, and mE of each antimicrobial–organism combination as described in the Materials and methods section.

3 The Enterobacterales group includes species: CFC, *Citrobacter freundii* complex; ECC, *Enterobacter cloacae* complex; EC, *Escherichia coli*; KA, *Klebsiella aerogenes*; KO, *Klebsiella oxytoca*; KP, *Klebsiella pneumoniae*; PM, *Proteus mirabilis*; PV, *Proteus vulgaris*; SM, *Serratia marcescens*.

Following the supplementary Etest comparison, the number of VME discrepancies decreased from 14 to 6, whereas the number of ME discrepancies decreased from 16 to 13. Trimethoprim/sulfamethoxazole accounted for the highest number of adjusted VMEs (3 of 6 total VMEs) and MEs (4 of 13 total MEs). Following supplementary analysis, VME rates for the Enterobacterales–piperacillin/tazobactam and Enterobacterales–ceftazidime combinations decreased to below the 3% threshold (Table 4; both 0%). The VME rate for the Enterobacterales–tobramycin combination improved modestly but remained above the 3% threshold (Table 4; 4.2%).

## Discussion

Within the local resistance epidemiology represented in this study, the VITEK REVEAL® system showed an overall agreement comparable to that of the SOC MicroScan platform based on EA and CA metrics. A major advantage of VITEK REVEAL® is its ability to generate phenotypic susceptibility results directly from positive blood cultures with an expected TAT of 8 h, assuming organism identification is available within the same timeframe using rapid identification methods such as the Verigene Gram-Negative Blood Culture Test, BioFire Blood Culture Identification Panel, Cobas ePlex Blood Culture Identification Panels, or direct MALDI-TOF identification. In comparison, conventional AST using MicroScan required an expected TAT of 16–24 h, in addition to approximately 16–18 h required for subculture recovery prior to AST setup (Figure 2). Earlier susceptibility reporting may have important implications for antimicrobial stewardship, including earlier de-escalation of broad-spectrum therapy, reduced drug toxicity, and more rapid optimization of targeted therapy in patients with bloodstream infections and sepsis.

Consistent with prior studies, VITEK REVEAL® achieved >90% CA and EA for the majority of antimicrobial–organism combinations, supporting its applicability in routine clinical microbiology laboratory workflows (Bui et al., 2025; Menchinelli et al., 2024; Simmons-Williams et al., 2026; Squitieri et al., 2025; Girlich et al., 2024). Lower CA was observed for amoxicillin/clavulanate and ampicillin/sulbactam, largely driven by mEs among Enterobacterales, particularly *E. coli*. These discrepancies are likely due to breakpoint proximity, biological variability associated with *β*-lactam/β-lactamase inhibitor combinations, and methodological differences between testing platforms. Notably, EA remained high (>94%) for all antimicrobial agents, indicating a close agreement in measured MIC values despite occasional categorical differences.

According to the CLSI, FDA, and European Committee on Antimicrobial Susceptibility Testing (EUCAST) criteria, acceptable AST performance includes VME and ME rates ≤3% and mE rates ≤10% (Humphries et al., 2018; FDA, 2009; Turnidge et al., 2025). The majority of antimicrobial–organism combinations met these criteria; however, several combinations exceeded recommended thresholds, including Enterobacterales tested against amoxicillin/clavulanate, ampicillin/sulbactam, piperacillin/tazobactam, ceftazidime, tobramycin, and trimethoprim/sulfamethoxazole. These elevated rates were likely influenced by small denominators and isolates with MIC values near interpretive breakpoints. A supplementary Etest analysis demonstrated that VME discrepancies showed similar concordance with both testing systems, whereas the majority of ME discrepancies were concordant with the MicroScan system. These findings suggest that many discrepancies occurred near categorical breakpoints rather than indicating substantial differences in MIC values between the systems.

Following supplementary Etest analysis, reductions in VME and ME discrepancies were observed for certain antimicrobial–organism combinations, whereas others showed minimal changes. Notably, VME rates for Enterobacterales tested against piperacillin/tazobactam and ceftazidime decreased to within acceptable performance thresholds, suggesting that VITEK REVEAL® results were concordant with Etest results. In contrast, the Enterobacterales–tobramycin combination showed VME rates above the acceptable threshold despite modest improvement, which may be due to a drug-specific limitation or the small number of resistant isolates included in the study. Similarly, trimethoprim/sulfamethoxazole accounted for a disproportionate number of residual VMEs and MEs following the supplementary analysis, highlighting challenges associated with susceptibility categorization near interpretive breakpoints.

Importantly, Etest was used as a supplementary exploratory tool rather than a reference adjudication method. Since reference BMD was not performed, these supplementary analyses should be interpreted as supportive observations rather than definitive discrepancy resolution.

CA reflects agreement in clinical susceptibility categorization, whereas EA evaluates MIC concordance within ±1 doubling dilution. Since small MIC shifts near clinical breakpoints can alter categorical interpretations and potentially influence antimicrobial selection, the assessment of both CA and EA provides complementary information. In this study, the majority of antimicrobial agents showed high CA, and all agents showed high EA, indicating generally strong agreement in MIC measurements despite occasional categorical discrepancies.

The current VITEK REVEAL® configuration is limited to a subset of GN organisms, a restricted antimicrobial menu, and testing directly from positive blood culture bottles. These limitations contrast with some rapid phenotypic AST approaches capable of testing both GN and Gram-positive organisms from blood culture bottles or isolates (Xue et al., 2025). Consequently, VITEK REVEAL® may not fully replace conventional automated AST platforms such as VITEK 2 and MicroScan. Instead, its most appropriate role may be as a complementary system that provides early, targeted susceptibility results in suspected bloodstream infections, analogous to rapid molecular blood culture diagnostics that provide early organism identification and resistance gene detection. Operational factors will also influence its clinical impact. Laboratories without 24-h staffing or integrated antimicrobial stewardship support may not fully realize the benefit of shortened TAT, as delays in result review and communication can reduce real-time clinical utility (Moore et al., 2023).

Cost-effectiveness is another important consideration. Although faster results may improve clinical management, the economic value of implementing VITEK REVEAL® remains unclear without formal clinical outcomes and cost analyses. Future studies should evaluate its impact on clinical outcomes and healthcare utilization, including length of hospital stay, antimicrobial use, and overall healthcare expenditures, to better define its role in routine practice (Galar et al., 2012; Roth et al., 2022; Booton et al., 2024).

In this study, 37 of 237 specimens (15.6%) were excluded because they did not meet eligibility criteria, representing a potential reagent cost consideration for laboratories (Figure 1). The majority of the excluded specimens (36/37; 97.3%) contained either polymicrobial cultures or non-target organisms. Since VITEK REVEAL® testing should be initiated within 16 h of blood culture positivity, there may be limited time to obtain conventional culture-based organism identification results before AST setup (Figure 2). However, rapid organism identification directly from positive blood cultures, including multiplex PCR panels or direct MALDI-TOF identification, may facilitate earlier recognition of ineligible specimens and reduce unnecessary testing. Integration of rapid identification methods with rapid phenotypic AST platforms may therefore improve both clinical utility and cost efficiency.

This study has several limitations. As a single-center evaluation, the isolate distribution, resistance epidemiology, and laboratory workflow reflect local practices, which may limit generalizability to other institutions. Reference BMD was not performed, and the comparator system consisted of a commercial automated AST platform rather than a reference method. Consequently, the findings should be interpreted cautiously and within the context of a comparative real-world clinical laboratory evaluation rather than a formal analytical validation study. The study was designed primarily to assess comparative performance within a real-world clinical laboratory workflow. Although comparison with a routine SOC AST platform may provide useful implementation and workflow data, it is not sufficient to establish definitive analytical performance characteristics of a new AST system. In addition, because the study relied on a non-reference comparator and included off-scale MIC comparison adjustments, a comprehensive bias analysis was not feasible. Larger multicenter studies incorporating geographically diverse isolates, expanded resistance phenotypes, and standardized reference methods are needed to further characterize the performance and clinical utility of the VITEK REVEAL® system.

## Data Availability

The original contributions presented in the study are included in the article/Supplementary material, further inquiries can be directed to the corresponding author.
