# Supplementary material for: Single-center clinical laboratory evaluation of VITEK REVEAL for rapid phenotypic antimicrobial susceptibility testing of Gram-negative organisms directly from positive blood cultures
Source: Front Microbiol. 2026 Aug 5;17:1890096. doi: 10.3389/fmicb.2026.1890096 (PMC13488308; doi:10.3389/fmicb.2026.1890096)
Supplement: Supplementary file 1 [file Table_1.docx]

**Supplementary Table 1**: Available antimicrobial drug and organism interaction list

|  |  | **Organisms** | | | | | | | | | | | | |
| --- | --- | --- | --- | --- | --- | --- | --- | --- | --- | --- | --- | --- | --- | --- |
|  |  | *Acinetobacter baumannii complex* | *Citrobacter freundii complex* | *Citrobacter koseri* | *Enterobacter cloacae complex* | *Escherichia coli* | *Klebsiella aerogenes* | *Klebsiella oxytoca* | *Klebsiella pneumoniae* | *Proteus mirabilis* | *Proteus vulgaris* | *Pseudomonas aeruginosa* | *Serratia marcescens* | |
| **Antimicrobial Drugs** | Amoxicillin/Clavulanate (AMC) |  |  |  |  | X^1^ |  | X | X | X |  |  |  |  |
|  | Ampicillin/Sulbactam (SAM) |  |  |  |  | X |  | X |  | X |  |  |  |  |
|  | Piperacillin/Tazobactam (TZP) |  |  | X |  | X |  |  | X |  | X |  |  |  |
|  | Ceftazidime (CAZ) | X |  | X | X | X | X | X | X |  |  |  |  |  |
|  | Ceftriaxone (CRO) |  |  |  | X | X | X | X | X | X |  |  |  |  |
|  | Cefepime (FEP) |  |  | X | X | X | X | X | X |  |  | X |  |  |
|  | Ceftolozane/Tazobactam (CFT) |  |  | X | X | X | X | X |  | X | X | X |  |  |
|  | Ceftazidime/Avibactam (CZA) |  | X | X | X | X | X |  | X | X |  | X |  |  |
|  | Ertapenem (ETP) |  |  |  |  | X |  |  | X | X | X |  |  |  |
|  | Meropenem (MEM) | X |  |  | X | X |  |  | X | X | X | X | X |  |
|  | Imipenem (IMP) | X |  | X | X | X |  | X | X |  |  | X | X |  |
|  | Meropenem/Vaborbactam (MEV) |  | X | X | X | X | X | X | X | X |  |  |  |  |
|  | Ciprofloxacin (CIP) |  | X |  | X | X | X | X | X |  | X | X | X |  |
|  | Levofloxacin (LVX) |  | X | X | X | X | X | X | X | X | X | X | X |  |
|  | Tobramycin (TOB) |  | X | X | X | X | X | X | X | X |  | X | X |  |
|  | Trimethoprim/Sulfamethoxazole (SXT) |  |  |  |  | X | X |  | X |  |  |  |  |  |

^1^The specific drug-bug combination was evaluated by bioMérieux VITEK REVEAL and MicroScan platforms
